# Death of Dr. R. W. Hynds

**Published:** 1905-09

**Authors:** 


### DEATH OF DR. R. W. HYNDS.

Dr. Robert W. Hynds, of Atlanta, died Friday, August 18th, as a result of typhoid fever, with which he had been suffering for six weeks.

The death of Dr. Hynds came as a profound shock to his friends, for, although he had been ill some time, it was not generally known that his condition was critical until Friday, when it was announced that he was dying.

He was a graduate of Vanderbilt University, at Nashville, Tenn., where he received his medical degree in 1900. Although a young man, Dr. Hynds was able and progressive and possessed an amiable disposition that readily won friends for him and made him admired by all who knew him.

At the time of his death Dr. Hynds was Assistant Bacteriologist and Chemist of Atlanta and Assistant Secretary of the State Board of Health, both of which positions he had held since the offices were created. He was also a member of the Fulton County Medical Society, and was on the staff of the Wesley Memorial Hospital.

---

THE Muscogee County Medical Society was organized at the court house, with the leading physicians of Columbus and vicinity as members. The doctors have had no organization in recent years, and the step taken to-night was regarded as a very timely one. Dr. W. L. Fitts, Counselor or Organizer for the Fourth District, supervised the details of organization. The action of the Muscogee county physicians in organizing is but in line with the general movement through the State. Membership in a county medical society carries with it membership in the State Medical Society, and eligibility to membership in the American Medical Association.

